# Miscellaneous Notices

**Published:** 1855-04-01

**Authors:** 


### MISCELLANEOUS NOTICES.

*Bethlem Hospital.*—We have read with much pleasure the last report of this hospital. It appears that on the 1st of January, 1854, there were (including those out on leave) 329 patients in the hospital, of whom 176 were males and 153 females. From this date until the 31st December 1854, there were 232 admitted, 218 discharged, and 21 died; leaving in the establishment on the 1st of January, 1855, the following 317, with 5 out on leave:—

| | Males. | Females. | Total. |
|---|---|---|---|
| Curable . . . . . . . . . . . | 58 | 79 | 137 |
| Incurable . . . . . . . . . . | 38 | 36 | 74 |
| Criminal. . . . . . . . . . . | 88 | 18 | 106 |
| | 184 | 133 | 317 |

The number of admissions during the year was below the usual average. The average of those reported as cured is $50\frac{8}{10}$ on the admissions. The health of the patients during the past year has been remarkably good. Dr. Hood makes some sensible observations in reference to the employment of the criminal lunatics. He says the marked effect of constant recurring occupation on the bodily health is only equalled by the improvement in the mental condition of the patients. We regret that, owing to this report only having just reached us, we are unable to quote more extensively from its pages. It is well and carefully drawn up, and embodies many points of deep interest. In our annual summary of the reports of British asylums, we shall again refer to this document.

*The American Journal of Insanity* continues its onward career of usefulness. The papers published in this periodical are always of great practical value. In an early number we propose doing ourselves the pleasure of analysing, at length, the last five or six numbers of this journal. We hope, in this article, to make amends for all our past omissions, *quoad* this able magazine. Want of space has alone prevented our doing so before.

*On the Construction, Organization, &c., of Hospitals for the Insane.* By Thomas S. Kirkbride, M.D., Physician to the Pennsylvania Hospital for the Insane, Philadelphia, 1854. This is an able, valuable, and deeply interesting work, from the pen of an eminent physician, who has a thorough practical knowledge of the subject-matter of the book. We have only space in this number to direct the attention of our readers to this essay. In our next publication we intend to review Dr. Kirkbride's work in detail, and to quote extensively from its pages.

*Letters to His Excellency Governor Manning on the Lunatic Asylums.* By Dr. H. Tregevant, M.D., Columbia, S. C., 1854. We postpone our critical remarks on this deeply interesting series of letters until our next number, when it will be reviewed in conjunction with Dr. Kirkbride's essay. The pamphlet has only just reached our hands.

*Insanity in Italy.* The observations of Dr. J. M. Galt (of America), and of Dr. Girolami, of Italy, relative to this subject will form the basis of an article that will appear in an early number. Dr. Girolami's books were unfortunately sent through the general post, and, in consequence of the extravagant postal charge demanded, we were reluctantly obliged to refuse the parcel. Many American periodicals, alas ! meet with the same fate. It occasionally happens that four and five shillings are asked for magazines, the selling price being only one and two shillings ! All books and periodicals should be sent through a London bookseller. Pamphlets and books are occasionally entrusted to private hands, and they consequently find their way into the post office. Hence their non-delivery, in consequence of their being charged *as letters.*

